# Pain Management in Intensive Care Unit Patients: The Role of Ultra-Short Acting Opioid: Remifentanil

**DOI:** 10.5812/aapm.10299

**Published:** 2013-07-01

**Authors:** Zahid Hussain Khan

**Affiliations:** 1 Department of Anesthesiology and Intensive Care, Imam Khomeini Medical Center, Tehran University of Medical Sciences (TUMS), Tehran, Iran

**Keywords:** Pain, Intensive Care Units, Remifentanil

It is an established fact that patients in the Intensive Care Unit (ICU) experience a plethora of events such as lack of sleep, anxiety, fear, disturbances caused by the continuous and menacing noises of the ventilators, machines, trolleys and above all, pain caused by innumerable reasons such as the underlying disease, suctioning of the endotracheal tube or invasive procedures including arterial punctures for blood gas analyses, cannulation of the central veins for fluid therapy, tracheostomies and insertion of chest tubes etcetra etcetra. of over whelming concern is pain which seems to be rather neglected despite numerous protocols and strategies apparently aimed at providing pain relief and anxiolysis to facilitate mechainical ventilation and therapeutic and diagnostic interventions. Pain management likely has attracted less attention in the critically ill patients in the ICUs owing to the fear of the cumulative effect and subsequent delayed recovery from the traditionally employed opioids and sedatives in this particular setting. The fear prevailing among the care givers seems to be justified on the grounds that critically ill patients have organ impairment of some degree thus delaying metabolism and excretion of drugs. This scenario ushers in problems of weaning from mechanical ventilation and extubation. There is a general consensus that effective analgesia and sedation is of paramount importance for patients in ICU in controlling pain, relieving anxiety and aiding compliance with mechanical ventilation thus providing patient comfort and patient satisfaction. In the past, besides analgesics and sedatives, neuromuscular blocking drugs (NMBD) were part and parcel of the armamentarum utilized in ICUs. Vecoronium was considered to be the choice as it lacked cardiovascular effects but its active metabolite- 3 desacetylvecoronium- could still accumulate in patients with renal impairment. Today the use of NMBD has been waning and presently their use is limited to some special situations such as intractable raised intracranial pressure, status epilepticus and some other conditions. Among the long acting opioids, recommended by the consensus conference, morphine is the most widely used analgesic agent in the ICUs worldwide (1). Despite the recommendation about the use of intravenous analgesic and sedatives in ICU patients, different protocols and combinations are being practiced (2). Combination of an opioid such as fentanyl or morphine for analgesia and a hypnotic agent such as propofol or midazolom for sedation is most commonly used (3, 4). However, there is a growing fear that the conventional or traditional opioids exhibit a prolonged effect as a result of redistribution and accumulation specially when given in repeated injections for days at a stretch or as infusions. Their effects become all the more protracted in the ICU patients most of whom have some degree of organ impairment. Concomittant organ/organs dysfunction adversely affect the metabolism of these opioids and exceedingly prolong their elimination thus offsetting the cherished goals of rapid weaning and timely extubation. These events in turn prolong the ICU stay causing a tremendous economic burden on the exchequer. Extension of stay in the ICU also brings in psychological stress and anguish for the family members besides inculcating an inevitable gloom and fear that the patient might be on a downhill course. No degree of counseling can appease the family members as long as they observe the patient being mechanically ventilated but in agony. The pharmaceutical industry has taken giant leaps in the recent past by introducing ultra-short acting opioids such as remifentanil. Remifentanil unlike the other opioids has a very rapid onset of action of about 1 min and quickly achieves a steady state. It is metabolized in an organ – independent fashion by non-specific esterases in the blood and tissues (5, 6). This latter property makes it unique among the existing opioids and makes it a particularly interesting option for the treatment of procedural pain in critically ill patients (3, 7). Remifentanil using target controlled Infusion (TCI) has also been found to be safe and effective in spontaneously breathing critically ill patients undergoing flexible fiberoptic bronchoscopy (8). There are two main treatment paradigms named the sedative – based approach and the analog-sedation technique which are currently in vogue to achieve analgesia and sedation in the ICU patients. These two strategies differ mainly in their primary goal. The universally proclaimed strategy till very recent past had been the sedative-based approach which revolves round the philosophy that the first line of treatment for ICU patients is sedation by titrating propofol or midazolam, and opioid analgesics co-administered with the sole aim of meeting the patient's perceived pain requirements. A pitfall with this technique is that the pain component may not be truly or objectively assessed and as such the analgesic requirements of the patient may be grossly underestimated. This is but natural as the main focus of this technique is to aim at adequate sedation. Another limitation of this technique is accumulation of the opioids if provided as boluses or as infusions for days and weeks. The second widely acclaimed strategy is the analgo-sedation technique which appears to be more appealing as it upholds relief of pain as its primary aim and providing sedation as prorenata or as needed or as anticipated per patient's perceived pain-relief requirements. Studies have shown that analgosedation is a well-tolerated approach for management of ICU sedation with improved patient outcomes compared to sedative – hypnotic approaches (9). In the sedation – based approach, morphine, fentanyl or sufentanil are commonly employed whereas in the analgo –sedation technique, remifentanil has been overwhelmingly advocated. Naturally, a technique which guarantees speedy recovery and rapid weaning is the best option but there are a score of problems when a choice has to be made. Ideal ICUs as are seen in the West and affluent countries are governed and directed according to internationally accepted guidelines i.e. one intensivist catering for a maximum of 7-8 patients, one nurse per patient and all amenities and accessories easily and freely available. In such ICUs, drugs such as remifentanil and propofol which need perfusers and a vigilant watch can be easily tailored to the patients’ needs. Besides, there are qualified and trained experts in ventilators who keep an eagle’s eye on the ventilatory parameters round the clock and conduct weaning and extubation as required and needed. In addition, the sedation agitation score and pain intensity scale scores are to be regularly monitored to achieve the optimal scores lest a patient is pushed into the realms of over sedation and coma or else left neglected with unremitting and agonizing pain coupled with agitation. All these tasks need round the clock supervision by trained personnel otherwise not only weaning from the ventilator and ICU stay will not be shortened but the morbidity and mortality would also exhibit a spiral hike. On the other hand, there are ICUs in the less privileged or developing world which are kept running with scanty means, less staff and less personnel. Acquisition of drugs such as remifentanil and propofol which besides being exorbitantly costly need gadgets and perfusers to be given in the exact prescribed dosages and concentrations apparently appear to be luxury items for treatment and as such are never or perhaps seldom used. On top of that the entry of drugs which play a pivotal role in the treatment strategy of a patient is banned owing to sanctions and nil bank transactions in some countries. Under such circumstances, the choice of a drug is beyond your imagination and comprehension and all you can do is to make your way through the inhospitable terrain with whatever stock or clothing you have at your disposal. Under the duress of circumstances, at times you say no to an easy option and yes to a difficult option. Thus, although remifentanil possesses sterling properties such as rapid onset and offset of action and devoid of any cumulative effects that can be of tremendous importance to the ICU patients, but its high cost and the extra personnel and appliances that are needed for its use make it a less attractive choice for use in ICUs lying in the far flung areas of the developing and the under developed world. Moreover, the safety and efficacy of remifentanil has not been investigated in critically ill patients beyond the 72 h period as reported by Breen et al. (3). They concluded that the time to offset of the pharmacodymic effects of remifentanil was statistically significantly longer in the moderate/severe group at the 24 h and 72 hr scheduled down titrations, and overall a greater variability existed in the times to offset of effects in this group compared with the normal/mild group. The differences in the normal and the renal impaired group could be due to the decreased clearance of remifentanil acid, a metabolite of remifentanil. Whether the curtailed clearance of remifentanil acid could add to the problem or could be a problem with the patient with renal impairment receiving long term remifentanil is a question that can only be answered with certainty if future trials are conducted. It has been shown that in deeply sedated patients receiving analgesic support based on a scale score, additional administration of remifentanil is not warranted for acute pain control assessed by Biepectral Index (BIS) variation (10). However, this hypothesis has not been validated in patients who need less sedation and thus definite conclusions cannot be drawn. In conclusion, the evolving pharmaceutical industry provides us with a variety of novel drugs and the fast growing industry provides us with new appliances, gadgets and tools such as automatic perfusors and TCI devices besides many other toys to use and play with, but using these paraphernalia should be tailored to the country's resources and income.
